# Gastroenteropancreatic Neuroendocrine Tumors in Multiple Endocrine Neoplasia Type 1

**DOI:** 10.3390/cancers4020504

**Published:** 2012-05-07

**Authors:** Francesco Tonelli, Francesco Giudici, Francesca Giusti, Maria Luisa Brandi

**Affiliations:** 1 Department of Clinical Physiopathology, Surgical Unit, Medical School, University of Florence, Largo Brambilla n° 3, Florence 50134, Italy; E-Mail: francesco.giudici1@gmail.com; 2 Department of Internal Medicine, Medical School and Regional Centre for Hereditary Endocrine Tumors, University of Florence, Largo Brambilla n° 3, Florence 50134, Italy; E-Mails: francesca.giusti1@alice.it (F.G.); m.brandi@dmi.unifi.it (M.L.B.)

**Keywords:** gastro-entero-pancreatic neuroendocrine tumors, MEN1, surgery, insulinoma, gastrinoma

## Abstract

We reviewed the literature about entero-pancreatic neuroendocrine tumors in Multiple Endocrine Neoplasia type 1 syndrome (MEN1) to clarify their demographic features, localization imaging, practice, and appropriate therapeutical strategies, analyzing the current approach to entero-pancreatic neuroendocrine tumors in MEN1. Despite the fact that hyperparathyroidism is usually the first manifestation of MEN1, the penetrance of these tumors is similar. They are characterized by multiplicity of lesions, variable expression of the tumors, and propensity for malignant degeneration. Both the histological type and the size of MEN1 neuroendocrine tumors correlate with malignancy. Monitoring of pancreatic peptides and use of imaging exams allow early diagnosis and prompt surgical treatment, resulting in prevention of metastatic disease and improvement of long-term survival. Surgery is often the treatment of choice for MEN1-neuroendocrine tumors. The rationale for surgical approach is to curtail malignant progression of the disease, and to cure the associated biochemical syndrome, should it be present.

## 1. Introduction

MEN1 syndrome is inherited in an autosomal dominant manner; the gene known to be associated with this syndrome is the *MEN1* gene, identified in 1997, and located on chromosome 11q13 [1]. This gene consists of 10 exons encoding a novel protein of 610/615 amino acid referred to as menin [2]. Very rarely, the mutation of the *p27^Kip1^(p27)/CDKN1B* gene has been implicated in MEN1 syndrome [3].

There are two forms of MEN1: familial and simplex form. The simplex form is characterized by only one affected individual within a family with no history of the disease, and occurs in 10% of cases. The familial form occurs with a significantly higher frequency, about 90% of cases, and is defined in an individual who has at least one first-degree relative with one or more main endocrine tumors, or involvement of only one organ and a MEN1 disease-causing germline mutation [4].

MEN1 syndrome is characterized by the occurrence of varying combinations of more than 20 endocrine and nonendocrine tumors, and can be defined by the presence of two “classic” endocrine tumors: parathyroid, pituitary, and neuroendocrine tumors of the gastro-entero-pancreatic tract, in an affected subject [5,6]. The MEN1 gene mutational analysis has identified, to date, over 1,336 mutations, germline and sporadic [7]. The mutations mainly involve, in more than 75% of cases, the absence of all or part of the protein product. A correlation between gene mutation and clinical behavior syndrome resulting in limitation of the use of this information in clinical practice has not been described. However, the mutational study allows early detection in still asymptomatic carriers. This allows the establishment of early and accurate clinical screening in such subjects to reveal, as soon as possible, the biochemical abnormalities that are associated with hormone-secreting endocrine neoplasia [8].

## 2. Entero-Pancreatic Endocrine Tumors

### 2.1. Epidemiology

The neuroendocrine tumors arising from pancreatic islet cells could be non-functioning (NF) characterized by variable production and release of neuroendocrine polypeptides without a specific syndrome and often showing a malignant course, or functioning with production of active hormones such as gastrin, insulin, vasoactive intestinal polypeptide (VIP), glucagon, and somatostatin leading to a clinical syndrome [9,10]. Endocrine tumors can also arise in the duodenum (Table 1) [9,10,11].

**Table 1:** Gastroenteropancreatic neuroendocrine tumors in multiple endocrine neoplasia type 1: penetrance, site and malignancy [9,10,11].

Tumor (Penetrance %)	Site	Malignancy (%)
Non-functioning (60–100)	Pancreas	64–92
Insulinoma (21)	Pancreas	12–20
Gastrinoma (50)	Pancreas Duodenum (>80%)	60
Glucagonoma (3)	Pancreas	35
Somatostatinoma (1)	Pancreas	70
	Duodenum/jejunum (44%)	
VIP-oma (1)	Pancreas Duodenum (10%)	40
GHRH-oma (1)	Pancreas	30

As opposed to their sporadic counterparts, MEN1-neuroendocrine tumors are characterized by early onset, multiplicity of lesions, variable expression of the tumors, and frequent propensity for malignant degeneration [9]. Small endocrine tumors, a finding that has been referred to as microadenomatosis, are generally present in all the pancreas. The pancreatic microadenomas are often accompanied by one or more macrotumors (diameter >5 mm), some of which may be functionally active [10,11].

In MEN1 syndrome, the penetrance of these tumors is very high, reaching 80–90% at 60 years of age, similar to that of parathyroid tumors, however, hyperparathyroidism is usually the first manifestation [9,10,11].

According to Ito *et al*. the rates of association of MEN1 and pancreatic neuroendocrine tumors differ between Eastern and Western nations, especially for nonfunctioning gastroenteropancreatic neuroendocrine tumors [12]. Both the histotype of MEN1 neuroendocrine tumor and the size correlate with malignancy [13].

Changes to the pathological classification of neuroendocrine tumors have recently been published [14]. The European Neuroendocrine Tumor Society (ENETS) developed guidelines to standardize and improve their diagnosis and therapy. According to the guidelines of the ENETS and of the World Health Organization (WHO), the pathology classification of gastroenteropancreatic neuroendocrine tumors consists in histological distinction between well and poorly differentiated tumors, proliferation-based grading, and TNM staging [14,15]. Based on these new criteria, a better prognostic stratification could be performed.

The management of MEN1-neuroendocrine tumors is still controversial [11]. The rationale for an aggressive surgical approach when such neoplasms are diagnosed is to curtail the malignant progression of the disease, and to treat the associated biochemical syndromes [16,17].

### 2.2. Diagnostic Tools

Localization imaging is of great importance, since surgical indication also seems to be based upon dimensional parameters. However, ultrasonography (US), Computed Tomography (CT), and/or Magnetic Resonance Imaging (MRI) cannot always provide satisfactory results in diagnosing neuroendocrine tumors, and furthermore, the diagnostic path for these lesions in MEN1 is not clear in literature. Preoperative US, MRI, or CT rarely localize lesions less than 1 cm, therefore missing most smaller MEN1-neuroendocrine tumors [18,19,20]. Somatostatin receptor scintigraphy (SSRS) is also an efficient tool in diagnosing neuroendocrine tumors, but its sensitivity depends on the number and the type of somatostatin (SS) receptors, and on the dimension of the tumor. Gallium-labelled somatostatin analogue peptides (DOTA-TOC, DOTA-TATE, DOTA-NOC) display good affinity for several types of SS receptors, and they have recently been employed because of their sensitivity in localizing lesions with a low expression of receptors type 2 and 5 [21,22].

Endoscopic ultrasound (EUS) seems to emerge as the most useful diagnostic tool because of its high sensitivity (up to 90%), even if it is strongly operator dependent [23,24,25]. According to recent studies, EUS seems to be the procedure of choice to detect neuroendocrine tumors with high sensitivity, even for lesions about 5 mm in diameter [26,27,28,29,30]. Furthermore, EUS is useful in preoperatively clarifying the relationship between pancreatic tumors and the main pancreatic duct. In MEN1 insulinoma, the possible simultaneous presence of multiple pancreatic nodules makes it difficult to localize the lesion responsible for the increased insulin overproduction. Percutaneous portal venous sampling has been used for the diagnosis of functioning pancreatic neuroendocrine tumors; however, it is an invasive technique, requiring a high level of expertise, and it may result in misleading information [31]. For MEN1 gastrinomas and insulinoma, the Selective Arterial Secretagogue Injection (SASI) test, and the selective intra-arterial calcium stimulation test with hepatic venous sampling for the dosage of gastrin and insulin/*C*-peptide, represent a valuable tool in regionalizing the area of gastrin and insulin secretion respectively, even when other diagnostic techniques do not visualize any mass, as frequently happens in duodenal gastrinoma [20,32]. The SASI test was shown to have a high predictive value in the Japanese experience, but similar accuracy was not reproduced in other studies [33].

Although no prospective randomized trial has been conducted on the validity of EUS in monitoring MEN1-neuroendocrine tumors, this imaging study seems to be the most accurate in baseline diagnosis or during the follow-up of patients.

## 3. Nonfunctioning Pancreatic Endocrine Tumors

### 3.1. General Tumor Characteristics

The widespread implementation of screening programs has led, more frequently than in the past, to the diagnosis of nonfunctioning tumors, with recent studies indicating that these tumors are the most common gastroenteropancreatic tumor associated with MEN1 [34,35,36].

One study identified 19% of MEN1 patients as having nonfunctioning PETs with an age-related penetration of 3, 34, and 53% at the ages 20, 50, and 80 years, respectively [34], and another study employing endoscopic ultrasonography has reported that 55% of asymptomatic MEN1 patients have nonfunctioning neuroendocrine tumors [35].

Cromogranin A (CgA) and neuron specific enolase (NSE) levels are frequently increased either before or after secretin stimulation test [37]. The natural history of MEN1-neuroendocrine tumors is not completely known, since few patients have been studied prospectively with a protocol based on high sensitivity diagnostic tools. It has been shown that when compared to the functioning tumors, the NF-neuroendocrine tumors are more frequently malignant, and their prognosis is worse [37,38,39,40]. This can be related to the larger size of these tumors by the time of the diagnosis [40]. Recent studies show that they are the most frequent leading cause of disease related death in MEN1 patients [16]. These aspects must be considered in planning follow-up, timing and type of surgery in these patients.

Gastrointestinal hemorrhage, biliary or gastric outlet obstruction, abdominal mass, and pain can be present at the time of diagnosis [41]. Metastasic disease is more prevalent in the liver than in the surrounding nodes [42]. Triponez *et al*. reporting the experience of the French Endocrine Tumor Study Group on 108 MEN1 patients, observed a correlation between the size of the tumors and their malignancy, with a critical diameter of 1.5–2 cm significantly marking the malignancy risk [35]. In the French experience, survival of the patients also correlated with the size of the MEN1 NF lesions [34,43]. Therefore, tumor size is an important prognostic factor for MEN1 NF-neuroendocrine tumors, influencing progression and patient survival.

### 3.2. Current Practice and Appropriate Therapeutical Strategies

Yearly follow-up with blood tests associated to appropriate imaging studies can be an acceptable option for monitoring both the increase of peptide values and the possible evolution of tumor size [42].

According to evidence shown by some authors, the simple increase of serial hormone levels is a reliable indication for surgery, considering the fact that asymptomatic patients with non-functioning tumors show a lower risk of metastatic disease and cancer-related death compared to patients operated on when symptoms were already present [44]. Other studies suggest the size as the best indicator for surgery, but the tumoral dimensional cut-off varies among different experiences [43,44,45,46,47,48,49,50,51]. Surgery is indicated when NF-neuroendocrine tumors larger than 1 or 2 cm in size are visualized [47,48,49,50,51]. Many retrospective studies have shown higher survival rates in surgically treated patients than in patients submitted only to medical care and follow-up [34,42,43,47,48,49,50,51].

The type of surgery for NF-neuroendocrine tumors is also controversial, but resection seems better than simple enucleation of the tumors [43,44,45,46,47,48,49,50,51]. Our policy is to indicate surgery when tumors approaching 2 cm in diameter are found, and to resect the most affected part of the pancreas, either the cephalic or the corporocaudal, at the same time enucleating tumors more than 5 mm in size present in the residual pancreas [52]. Lymphadenectomy of peripancreatic nodes, hepatoduodenal ligament nodes, and hepatic and splenic artery nodes, must be associated.

Total pancreatectomy is another surgical option for these patients, but given its intrinsic long term morbidity, it must be limited to cases with extremely diffuse and large tumors, or when the patient already suffers from insulin-dependent diabetes [53].

## 4. Insulinoma

### 4.1. General Tumor Characteristics

Sporadic insulinomas are neuroendocrine tumors arising from the pancreatic islet β-cells. Insulinomas are equally distributed within the pancreatic parenchyma, and they are ectopic in only 3% of cases. Since sporadic insulinoma is usually a solitary lesion, if multiple pancreatic nodules are identified, a MEN1 diagnosis should be considered [54].

Insulinoma is characterized by a prevailing and inappropriate secretion of insulin that causes a syndrome of hypoglycemia (100%), neuroglycopenic symptoms (80–100%), and symptoms of adrenergic stimulation (80–100%). It usually manifests with subtle symptoms such as hunger, fatigue, nausea, vomiting, paresthesia, peripheral neuropathy, and blurred vision. These symptoms are partly masked by the tendency of these patients to compensate for hypoglycemia by overeating, so these patients are often overweight. The most dramatic symptoms of hypoglycemia, such as neuropsychiatric manifestations, oscillating moderate personality changes, confusion, and coma, can lead to difficulties in diagnosis [55,56].

Hypoglycemic syndrome in MEN1 patients usually occurs in the second or third decade of life, one decade earlier than patients with sporadic insulinomas [57,58]. Insulinomas in MEN1 are generally benign (90%) and occur in about 15–20% of affected patients. Their size is variable from less than 5 mm to more than 2 cm. Insulinoma in MEN1 are uncommonly malignant with extrapancreatic infiltration and/or metastasis (2.4%–17.9% with an average of 8.4%) [54,57,58,59,60]. Insulinomas without signs of angioinvasion and showing a mitotic rate of Ki-67 <2% are to be considered benign [54,57,58,59,60].

### 4.2. Current Practice and Appropriate Therapeutical Strategies

Pancreatic insulinoma is characterized by fasting hypoglycemia with high plasma or serum concentration of insulin and *C*-peptide or proinsulin. However, hypoglycemia (<40 mg/dL) is found only in a minority of patients (less than 40% of cases) while inappropriate secretion of insulin is present in a higher percentage of patients. For these reasons, the fasting test is important in such cases, and that is, positive when the glycemia decreases to 40 mg/dL after 24–72 h of fasting. This test can also be associated with a mathematically calculated value of insulin/glycemia ratio which in the presence of insulinoma must be superior to 0.3 [55,56].

While indications for localization and treatment of sporadic insulinoma are well established, clear guidelines could not be drawn for the management of MEN1 insulinoma [5]. Prompt surgery for insulinoma can be pivotal, since hypoglycemia is not easily controlled by drugs. Different from sporadic insulinomas, enucleation or limited resection of pancreatic nodules in MEN1 patients may lead to the persistence of hypoglycemia [61]. Furthermore, the concomitant presence of other functioning and non-functioning tumors with potential malignancy must be considered in order to appropriately indicate the timing of surgery in MEN1 patients.

Even if enucleation is the treatment of choice for sporadic insulinoma, the surgical cure rates after enucleation in MEN1 insulinoma is only around 60% [54,57,62]. Therefore, when MEN1 insulinomas are located in the left pancreas, the proposed treatment should be distal pancreatic resection up to the superior mesenteric vein, associated with enucleation of any additional tumors in the pancreatic head. This type of treatment is accompanied by good results in terms of cure rates (about 90%) and with limited postoperative complications [54,63,64]. This procedure is justified for two reasons: to prevent the persistence and the recurrence of the hypoglycemic syndrome, and to avoid the development of other potentially malignant pancreatic tumors. Otherwise, when the pancreatic head shows major involvement, to guarantee the best surgical results, it is important to resect the more affected pancreatic side, even performing a pancreatoduodenectomy (PD). In literature, PD is rarely considered the standard of care for MEN1insulinoma, because of the potential surgical complications [52,65]. However, it is important to underline that overall morbidity and development of a pancreatic fistula are similar when comparing the different surgical procedures with expert surgical teams performing the surgery [33,43,62,63,64,65,66,67,68,69].

Intraoperative confirmation of complete resection of the insulin producing lesions could be useful to decrease the percentage of persistence or recurrence of hypoglycemic syndrome. Both intraoperative serum glucose and insulin levels have been investigated, and rapid insulin immunoassay, from peripheral blood samples, has been proven in the literature reliably indicating successful removal of sporadic insulinoma [70,71,72,73]. However, Proye *et al*. examined eight cases of MEN1 insulinomas and the intra-operative insulin assay correctly predicted the favourable outcome of surgery only in five of them [69]. In our experience, the concomitant monitoring of glycemia and insulin levels calculating the insulin/glucose ratio during the surgical pancreatic procedure made it possible to avoid misinterpretation of the intraoperative test and have an absolute relationship of this test with the final outcome in eight MEN1 patients [74].

## 5. Gastrinoma

### 5.1. General Tumor Characteristics

Zollinger Ellison Syndrome (ZES) is characterized by excessive incretion of gastrin and consequent hypersecretion of hydrochloric acid by oxintic cells of the stomach. Symptoms can include upper abdominal pain, esophageal reflux, peptic ulcers, and diarrhea. ZES affects at least 40% of MEN1 patients, representing 20% of all diagnosed gastrinomas. If the peptic ulcer is not properly diagnosed and treated, gastro-duodenal perforation, partial or complete gastric outlet obstruction, and hematemesis or melena may result [55,75].

Compared to the sporadic counterpart MEN1, gastrinomas are more prevalently located in the duodenum (80%–100% of cases) than in the pancreas [76,77]. In MEN1, duodenal gastrinomas are usually multiple, smaller than 5 mm in diameter, associated to foci of gastrin-positive cells, and with a predominance in the proximal duodenum, while the pancreatic counterpart is usually single and greater than 1 cm in diameter. At time of diagnosis, the pancreas presents several nodules of non functioning neuroendocrine tumors [33,52].

The diagnosis of gastrinoma is based on the finding of levels of gastrin superior than 100 pg/dL. However, elevated fasting gastrin values (also superior than 100 pg/dL) can be observed in patients with achlorhydria (patients on therapy with inhibitors of gastric acid secretion [Proton Pump Inhibitor (PPI)] or with autoimmune chronic atrophic gastritis). In these cases, the evaluation of gastric pH is clarifying. In doubtful cases, it is useful to perform the stimulation test with a bolus of secretin (2 µg/kg). An increase of gastrin >120 pg/dL after 2–5 min of secretin infusion is considered a suggestive result for gastrinoma; the response to secretin is relative to the presence of receptors for secretin on the gastrinoma cells. This test has a sensitivity and specificity of about 90% for detecting gastrinomas [6]. However, it is necessary to underline that this test can be positive also in patients with hypergastrinemia due to atrophic gastritis, in fact, it has been shown that antral G-cells have secretin receptors and release gastrin when stimulated with pharmacological doses of secretin [20,78,79,80,81].

### 5.2. Current Practice and Appropriate Therapeutical Strategies

ZES symptoms are well managed pharmacologically by Protonic Pomp Inibitors (PPIs), and complications from peptic disease are becoming rare [76,77]. As the site of gastrinomas is prevalently, but not exclusively, in the duodeno-pancreatic area (gastrinomas triangle), the site of the gastrinoma must first be located. Pre-operative (US, CT, SSRS-Octreoscan, EUS, SASI test) and intra-operative methods of localization, such as palpation, duodenal transillumination, and IOUS were tested, but showed limited usefulness, especially when tumors are of microscopic size, or when concomitant non functioning pancreatic neuroendocrine tumors are present. More recently, intraoperative gastrin measurements were offered as potentially capable of determining the extent of resection and its radicality [20,33].

Radical excision of gastrinomas is critical since it requires the removal of either duodenal mucosa or cephalic pancreas, procedures which present risk of severe operative complications.

Indications for surgical treatment are established at present for sporadic gastrinomas, but clear surgical recommendations cannot be drawn for the management of MEN1 gastrinomas in terms of timing and aggressiveness of surgery. Some Authors recommend explorative surgery as soon as ZES in MEN1 is diagnosed [82,83,84]. Others suggest surgery only when a 2–3 cm lesion is preoperatively localized, since a high risk of metastatic spread is reported for this diameter [33,77,85,86,87,88]. These controversies depend on the hypothesis that two different populations could exist in the group of MEN1-gastrinomas, one showing an indolent course even in the presence of metastatic disease (usually in peripancreatic nodes) and another showing rapid tumor progression with hepatic metastatization, independent of the size of the tumors [33,85,86]. However, no predictive factors have been identified so far to stratify these two populations of gastrinoma with a consequent different surgical approach.

Moreover, MEN1 related gastric carcinoids (types II and IV), secondary to chronic hypergastrinism, are described in up to 37% of MEN1 cases, independent of the size of the gastrinomas [33,88].

Markers of gastrinoma aggressiveness can be recognized in elevated fasting gastrin levels, poor tumoral differentiation, high mitotic number, Ki 67 positivity, and expression of progesterone receptors [88].

Finally, the extent of surgery itself is a matter of debate. Conservative surgery was suggested by longitudinal duodenotomy performing simple excision of duodenal gastrinomas less than 5 mm in size or duodenal full-thickness excision of lesions more than 5 mm in size, peripancreatic lymphadenectomy, excision of pancreatic cephalic macroscopic lesions, and a distal pancreatectomy if multiple macroscopic neuroendocrine tumors are present [82,89]. However, this approach was shown to result in a low cure rate for gastrinomas (especially for the biochemical cure) making recurrence more likely, and exposing patients to the consequences of chronic hypergastrinemia [33,88]. Even if a recent experience on few selected patients undergone Thompson procedure seems to achieve better results in term of postoperative eugastrinemia, it is not clear if it is due to selection of the patients or to the modality of the lymphadenectomy, that is more extended than in the previous reports [90]. On the basis of these considerations, as MEN1 patients can exhibit gastrinoma metastases with lack of clear-cut markers for tumor progression, some Authors have proposed earlier and more radical surgery [33,86,87,88]. Pancreatoduodenectomy (PD), together with regional lymphadenectomy, appear to be the best treatment, assuring long term oncological and biochemical cure [20,33,82,83,84,86,87,88,89,90,91,92].

PD is an aggressive procedure, considering the operative risks (pancreatic or biliary fistula, pancreato-jejunal anastomotic leak, pancreatitis and haemoperitoneum) and the adverse metabolic consequences. However, in the hands of experienced surgeons, postoperative mortality and morbidity of PD has been progressively reduced, becoming at present quite acceptable [52].

Imamura *et al.* [20] developed and performed a pancreas preserving total duodenectomy (PPTD) in MEN1 gastrinoma, but it seems to be indicated only when the pancreas is spared by macroscopic tumors. Furthermore, experience with this type of surgery is still limited.

## 6. Glucagonoma, Somatostatinoma, VIPoma, GHRHoma

### 6.1. General Tumor Characteristics

Glucagonoma, somatostatinoma, VIPoma, and GHRHoma rarely occur in MEN1. These tumoral entities usually localize in the pancreas in MEN1, even though VIPoma has been described in the duodenum, and somatostatinoma both in the duodenum and the jejunum. Most of the cases present with a tumor size of more than 3 cm in diameter and malignant behavior [93]. Clinical evidence is of great importance to promptly recognize these tumors.

Glucagonoma syndrome is referred to as the 4D syndrome for the association of dermatitis, diabetes, deep vein thrombosis, and depression. The typical form of dermatitis associated with glucagonoma syndrome is necrolytic migratory erythema, which consists of painful pruritic plaques that start in the abdomen and groin, and spread to the trunk and extremities. The cause is unknown, although it is postulated to result from a direct effect of glucagon, prostaglandin release, or a deficiency of amino acids or zinc. Other symptoms include diarrhea, glossitis, weight loss, various neurologic and psychiatric symptoms, mild or severe diabetes mellitus, and thromboembolism, most commonly deep vein thrombosis and pulmonary embolism. In normal conditions, the glucagon does not exceed 200 pg/mL, while in patients affected with glucagonoma, the hormonal concentrations are generally superior to 500 pg/mL [94].

Somatostatinoma is characterized by hyperglycemia (95% of cases) and hypochlorhydria (85%), and values of somatostatin in concentrations higher than normal. However, the clinical features of somatostatinoma syndrome are nonspecific, and therefore difficult to recognize, given that somatostatin inhibits intestinal absorption and release of insulin, glucagon, gastrin, and pancreatic enzymes, which may lead to diabetes mellitus, steatorrhea, diarrhea, chole-lithiasis, hypochlorhydria, and weight loss [94].

VIPoma syndrome, also known as Verner-Morrison syndrome, usually presents a Watery-Diarrhea-Hypokalemia-Achlorhydria (W.D.H.A.) syndrome [95]. The amount of diarrhea is often more than 6–8 L/die, even with fasting, and it leads to hypokalemia, hypochlorhydria, and profound dehydration. Weight loss, facial flushing, and abdominal pain may be also present. It is characterized by high plasma concentration of vasoactive intestinal polypeptide (VIP), as determined by immunoassay test (<75 pg/mL or <75 ng/L) [94].

Pancreatic GHRHoma is MEN1 related in about 10% of cases; it is characterized by elevated serum concentrations of growth hormone and growth hormone releasing hormone (GHRH), and the patients result affected with acromegaly [96].

### 6.2. Current practice and Appropriate Therapeutical Strategies

With regard to these diseases, unfortunately, there are no reliable provocative tests, and the blood value of these hormones is not always easy to define; for example, these proteins can easily go to meet proteolytic degradation [55,97,98].

Recommendations for diagnosis are systematic peptide biochemical assays followed by imaging exams in case of increased hormonal values. The diagnostic imaging instruments are the same, and associated with the same issues, as those described for NF-PETs (US, CT, MRI and EUS) [99].

Since clinically evident glucagonomas, VIPomas, somatostatinomas, and GHRHoma are rare in patients with MEN1, there is no consensus statement about their management and treatment [93,100].

Even in absence of clear surgical guidelines for these tumors, it seems important to consider surgery in the presence of these syndromes, especially when they are associated with a diagnosed lesion(s) more than 1cm in size. Surgical approaches have to respect the same oncological criteria previously described for NF and functioning MEN1-neuroendocrine tumors [93]. Intraoperative management of somatostatinoma, glucagonoma, VIPoma, and GHRHoma in MEN1 could take advantage of the same localization methods utilized for the other pancreatic neuroendocrine tumors previously described. The prognosis is poorer than for gastrinomas and insulinomas, with a 10-year survival rate of about 50%, similar to that of non-functional tumors. The poor prognosis seems to be related to the high rate of malignancy at the time of surgery. In this context is easy to understand the importance of prompt preoperative diagnosis.

## 7. Gastric Carcinoids

Gastric endocrine tumors are currently classified as: (I) well differentiated (94% of all neuroendocrine gastric tumors) argyrophilic lesions, mainly composed of ECL cells; and (II) poorly differentiated neuroendocrine carcinomas (5–6% of all neuroendocrine gastric tumours) [101]. The first group is divided into three distinct subtypes: type 1: gastrin-dependent type of carcinoid accompanied by chronic atrophic gastritis complicated with hypergastrinemia and G cell hyperplasia; type 2: associated with hypertrophic gastropathy and hypergastrinemia due to MEN1/ZES; and type 3: sporadic carcinoid [102]. The second group is diagnosed apart from the undifferentiated histology for the negative expression of the large isoform 2 of the human vesicular monoamine transporter (VMAT-2. Usually, gastric endocrine tumors in MEN1 syndrome encompass the type 2 well-differentiated lesions (smaller than 1 cm in diameter, multicentric, and with gastric wall invasion limited to the mucosa and submucosa) [100], but sometimes they present as the large, poorly differentiated carcinomas (VMAT-2 negative) that have recently been classified as type 4 carcinoids [102,103,104]. Even if the site of development is the fundic area, carcinoids type 2 can also arise in the antral gastric mucosa. This was explained by the growth in the antral mucosa of ectopic ECL cells [105]. Type 2 MEN1 gastric neuroendocrine tumors are associated with ZES and hypertrophic fundic gastropathy [102]. The mean age at diagnosis is 50 years. With time, almost all MEN1 patients affected by ZES develop a fundic argyrophil cell hyperplasia, and in a third of the cases gastric carcinoids [101]. Argyrophilia and immunoreactivity to general markers (CgA and synaptophysin), to cytosolic markers (neuronespecific enolase), and to specific markers (gastrin, somatostatin, serotonin, and VMAT-2) are used as diagnostic tools [106]. In a prospective follow-up (mean eight years) of 57 MEN1 gastrinoma patients, gastric carcinoids were reported in about 25% of the cases, being more frequently represented in patients affected by aggressive gastrinoma (about 60%) than in those with non aggressive gastrinoma (only 10%) [107].

Hypergastrinism is frequently associated with ECL hyperplasia, but an additional factor is probably required to promote the transformation into a carcinoid, as some other hypergastrinemic conditions, such as sporadic ZES and chronic antisecretive therapy, are not associated with gastric carcinoids [108,109]. Indeed, reduced MEN1 gene function represents a promoting factor towards transformation, as confirmed by loss of heterozygosity at 11q13 in gastric carcinoids [110,111,112]. These observations support the theory that these tumors are part of the neoplastic constellation of MEN1 syndrome, with hypergastrinemia playing only a growth-promoting role. The management of type 2 gastric carcinoids has to be approached in the context of the MEN-1 gastrinoma [103,113].

## 8. Therapy with mTor and Tyrosine Kinase Inhibitors

In two recent multicenter, randomized, double-blind, phase 3 trials, Everolimus, an oral inhibitor of mammalian target of rapamycin (mTOR), orally administrated at a dose of 10 mg once daily, and Sunitinib, a multitargeted tyrosine kinase inhibitor administered at the dose of 37.5 mg per day, were compared with placebo in 207 and 86 patients, respectively [114,115]. Patients were affected with advanced pancreatic neuroendocrine tumors in which the disease had progressed within the previous 12 months. Patients were eligible to be included in the studies if they were 18 years of age or older and had advanced (unresectable or metastatic) pancreatic neuroendocrine tumors and radiologic documentation of disease progression (an unequivocal increase in the size of tumors) in the 12 months preceding randomization. Everolimus, compared to placebo, improved progression free survival. The adverse events seen with this agent were mainly grade 1 and 2 events, thus allowing for long-term daily administration [114].

Sunitinib improved progression free survival, overall survival, and the objective response rate as compared to placebo [115]. It is not yet clear whether sunitinib and everolimus can be combined, and, if so, whether antitumor activity would be further increased with combined treatment. It has previously been shown that Everolimus can be safely administered to patients with neuroendocrine tumors either with or without concurrent octreotide long-acting release (LAR) therapy [116].

## 9. Conclusions

Functioning entero-pancreatic MEN1-neuroendocrine tumors characterized by hormonal hypersecretion syndromes should be operated upon at the moment of diagnosis. The evaluation of baseline hormonal value may be sufficient for their diagnosis, but it often becomes necessary to perform the stimulation test. The functional tests are very important since the high circulating levels of a given gastrointestinal hormone do not necessarily indicate the presence of cancer. Elevated levels of a hormone may also result in gastrointestinal proliferation of non-neoplastic cells, as in endocrine hyperplasia. The application of evocative and/or withdrawal tests becomes particularly useful in the differential diagnosis between hyperplasia and cancer. In fact, diffuse endocrine system cells respond appropriately to the regulation of exogenous stimuli; conversely, cancer cells respond in an autonomous and sometimes in a paradoxical way, thereby enhancing the diagnostic differentiation. Although ZES symptoms are pharmacologically well managed by PPIs, gastrinomas are frequently malignant, and chronic hypergastrinemia can induce the development of gastric carcinoids. In these tumors, an increase of gastrinemia values, and/or the dimensional growth of pancreatic gastrinoma/s during follow-up, can be considered signs of oncologic aggressiveness, and indication for surgery.

Frequently, NF neuroendocrine tumors in MEN1 are malignant. For this reason, the goal of screening in these patients is to identify MEN1-associated tumors at an early stage (around 1 cm diameter) and thereby reduce both the morbidity and mortality associated with these tumors.

The NF neuroendocrine tumors should be operated upon once the pancreatic lesion reaches 2 cm in diameter; yearly follow-up should be reserved for lesions smaller than 2 cm in size.

In MEN1 patients, surgery should be performed with resection of the most affected hemi-pancreas, with associated enucleation of >5 mm nodules if present in the residual pancreas.

PD, along with regional lymphadenectomy, appears to be the treatment of choice for radical treatment of MEN1 gastrinoma given the almost exclusive presence of gastrinoma in the duodenum. If surgery is controvert or not indicated, medical treatment of neuroendocrine neoplastic lesions is the therapy of choice with “long acting” somatostatin analogues (SSAs), as it appears to control the symptoms related to hormonal hypersecretion, and control or slow down the tumoral growth of neuroendocrine tumors.
